# Reply to Khan et al., Critical Insights: ABC score is a better predictor for 30-day mortality in upper gastrointestinal bleeding: A prospective single-center study

**DOI:** 10.1007/s12664-025-01750-2

**Published:** 2025-02-22

**Authors:** Vikas Pemmada, Athish Shetty, Ganesh Bhat

**Affiliations:** Department of Gastroenterology, Kasturba Medical College, Manipal, Manipal Academy of Higher Education, Manipal, 576 104 India

Dear Editor,

We appreciate the constructive feedback and critical insights on our original article, “ABC score is a better predictor for 30-day mortality in upper gastrointestinal bleeding: A prospective single-center study”, published in the *Indian Journal of Gastroenterology *[1, 2]. We would like to reply to the comments on our article.

Regarding research methodology, the period of 30-day mortality outcome has been widely studied in earlier studies using the ABC score along with the landmark multicentric study by Laursen et al. [3–5]. The 30-day mortality outcome has also been used in other gastrointestinal bleeding (GIB) scores like GBS, AIMS65 and pre-Rockall [6]. Also, a large meta-analysis on transfusion strategies, which included studies about acute blood loss and gastrointestinal bleeding, concluded that results of mortality analyses at hospital discharge, 90 days and long-term are consistent with the results for mortality at 30 days [7]. The data on long-term outcomes of GIB is scarce and often attributed to the presence of coexisting conditions and patients’ lifestyles, as mentioned in a recent study [8]. As our study was conducted in a tertiary referral centre, we often receive patients after partial management with blood transfusions and medications from elsewhere. To incorporate acute GIB patients and to maintain the homogeneity of the study, we have created a 48-hour window period in our inclusion criteria.

Blood transfusions were made to maintain a target of hemoglobin (Hb) ≥ 7 g/dL or higher in patients with clinical evidence of intra-vascular volume depletion or comorbidities such as coronary artery disease. This was described as the standard of care for all our patients in the study and we followed a restrictive transfusion strategy. Compared to a liberal transfusion strategy, a restrictive strategy significantly improved outcomes in patients with acute upper gastrointestinal bleeding [9]. We acknowledge the limitations of the level of albumin and mortality as there can be several confounding factors including comorbidity. However, albumin is an essential variable among the blood tests required for the calculation of the ABC score. Our study showed that hypoalbuminemia was one of the statistically significant factors in predicting mortality in upper gastrointestinal bleeding as mentioned in Table 4 [1].

The ABC score has been rigorously developed and validated in prior studies across diverse populations and settings. It was proposed by Laursen et al. in an international multicentric cohort study, which included 4019 patients with upper GIB in 2021 and is further validated by another study by Saade et al. in 2022 [3, 4]. So, the external validation cohort was not essential for our prospective study. Our study does not aim to create or validate the ABC score itself, but to demonstrate its superior predictive value for 30-day mortality in a specific clinical context for patients with upper gastrointestinal bleeding.

## Data Availability

Reply to correspondence.

## Declarations

### Disclaimer

The authors are solely responsible for the data and the contents of the paper. In no way, the Honorary Editor-in-Chief, Editorial Board Members, the Indian Society of Gastroenterology or the printer/publishers are responsible for the results/findings and content of this article.
